# Study partners: essential collaborators in discovering treatments for Alzheimer’s disease

**DOI:** 10.1186/s13195-018-0425-4

**Published:** 2018-09-27

**Authors:** Emily A. Largent, Jason Karlawish, Joshua D. Grill

**Affiliations:** 1 Department of Medical Ethics and Health Policy, University of Pennsylvania Perelman School of Medicine, Blockley Hall, Room 1403, 423 Guardian Drive, Philadelphia, PA 19104 USA; 2 Leonard Davis Institute of Health Economics, 3641 Locust Walk # 210, Philadelphia, PA 19104 USA; 3 Department of Medicine, University of Pennsylvania Perelman School of Medicine, 3400 Civic Center Boulevard, Building 421, Philadelphia, PA USA; 4 Department of Neurology, University of Pennsylvania Perelman School of Medicine, Dulles 3rd Floor, 3400 Spruce Street, Philadelphia, PA 19104 USA; 5 Department of Psychiatry and Human Behavior, University of California Irvine, Neuropsychiatric Center, 101 The City Drive South,Third Floor Orange, Irvine, 92868 USA; 6 Department of Neurobiology and Behavior, University of California Irvine, 2205 McGaugh Hall, Irvine, CA 92697 USA; 7 Institute for Memory Impairments and Neurological Disorders, Institute for Clinical and Translational Science, 2642 Biological Sciences III, Irvine, CA 92697-4545 USA

**Keywords:** Alzheimer’s disease, Clinical trials, Ethics, Study partners, Abbreviations, AD Alzheimer’s disease, FMLA Family and Medical Leave Act of 1993, FPL Federal poverty level, MCI Mild cognitive impairment, SES Socioeconomic status

## Abstract

**Background:**

Global leaders have set an ambitious goal of developing interventions to effectively treat or prevent Alzheimer’s disease by 2025.

**Case presentation:**

Achieving this goal will require clinical trials to test promising interventions, yet Alzheimer’s researchers are confronting a clinical trial recruitment crisis. One reason for this is that Alzheimer’s disease trials must enroll “dyads” composed of both a participant and his or her study partner.

**Conclusions:**

In this article, we argue that it is essential to identify ways to facilitate study partner participation, such as removing logistical barriers, offering payment, and providing paid, protected time off for study visits. Facilitating participation, particularly among non-spousal study partners, should offer a twofold benefit: faster accrual and greater generalizability of results.

Alzheimer’s disease (AD) is one of the costliest health-care challenges of our time. The costs are human and financial. Globally, more than 46 million people live with dementia, and the estimated worldwide cost of dementia is roughly a trillion dollars [1]. A disease-modifying drug could reduce these significant social and economic costs to patients, their families, and the global economy. Unfortunately, no such drug has been discovered. To change this, global leaders have set an ambitious goal of developing interventions to effectively treat or prevent AD by 2025 [2, 3]. Achieving this goal will require multinational clinical trials to test promising interventions.

Recruitment of study participants is among the most consistent, challenging, and costly barriers to trial success in any therapeutic area. AD is no exception [4]. AD researchers currently confront a recruitment “crisis” recognized as among the greatest obstacles to developing new interventions [5]. Recruitment difficulties prolong trials and increase costs. Trials that ultimately fail to reach their target enrollment are more likely to go unpublished given the likelihood that they will fail to meet the primary outcome. Even in trials that eventually succeed in enrollment, participants rarely represent patients with AD on the whole given disparities in age, race, and comorbidities [6–8]. The failure to recruit sufficient numbers of representative participants at an acceptable rate delays scientific progress, wastes financial resources, and squanders the contributions that participants make to research [9].

One reason for the AD recruitment crisis is that trials must enroll “dyads” composed of a participant and the participant’s “study partner” [10]. Study partners share in the decision-making process to join an AD trial. Once enrolled, they ensure trial compliance and act as a knowledgeable informant, reporting on the participant’s cognitive and functional status to help evaluate the intervention’s safety and efficacy. Being a study partner necessitates the commitment of time, effort, and insight into the research participant’s cognition and function. In short, being a study partner is work [11]. If an older adult doesn’t have someone who’s able or willing to do this work with her, then the study partner requirement is a barrier to her research participation.

Here, we argue that when a trial is being designed and conducted, the work of being a study partner should be assessed and justified to the greatest practicable extent. Furthermore, the benefits of being a study partner should be enhanced in order to promote recruitment and retention. The central premise for this argument is that we are currently relying on free labor to fix a free-labor problem. For instance, as much as half of the AD-related costs to the US, estimated from $157 to $215 billion per year, are the costs of informal caregiving provided by over 15 million American adults [12, 13]. Such informal caregiving is the hallmark of dementia care worldwide [1]. These caregiving costs are used to make the most compelling argument possible for the social burden of AD and so for the urgent need to discover interventions to prevent and treat it. Yet, at present, little is done to mitigate burden or increase benefits when asking individuals—typically caregivers in studies of symptomatic AD—to do the work of serving as a study partner in AD research. Rather ironically, these individuals are asked to take on even more unpaid work.

Slow recruitment and a persistent lack of representativeness in study populations suggest that this reliance on unpaid labor is not working well. By taking seriously the work of being a study partner, we can increase both the number and types of individuals serving as study partners.

## Background

AD is a neurodegenerative disease characterized by progressive and disabling cognitive impairments ultimately leading to death. Research into biomarkers of AD pathophysiology, including beta-amyloid plaques and neurofibrillary tangles of tau protein, has led to a conceptualization of AD as a “continuum” [14]. Researchers posit three stages of AD. In the “preclinical” stage, individuals have pathophysiologic changes identifiable by biomarker assays, yet they are cognitively and functionally intact. Preclinical AD is solely a research diagnostic construct, not currently used in clinical practice [15]. The second stage, mild cognitive impairment (MCI), is characterized by measurable changes in cognition that do not meaningfully affect daily functioning. Finally, dementia is characterized by cognitive decline that impairs and eventually precludes daily function.

AD trials now enroll patients in the preclinical, MCI, and dementia stages. Study partners are vital to trial success across each stage, although as the participant experiences cognitive decline, the role of the study partner changes and may become more time-consuming and labor-intensive. Consistent across all three stages, however, is the study partner’s responsibility to provide data about cognition and function, data that are essential to establishing the older adult’s trial eligibility and the value of a therapy. Although it is conceivable to design an AD trial that doesn’t require dyads, there would be significant limitations of such trials. Most notably, these trials would not be able to use traditional informant-based measures of patient function, which trials routinely use as co-primary outcome measures [16, 17].

Because AD trial participation is necessarily the work of a dyad, if an otherwise eligible individual cannot identify a study partner, he or she cannot enroll. For example, more than a quarter of registrants (333 out of 1202) in the UC Irvine Consent-to-Contact (C2C) Registry answered “no” when asked “Is there an individual who could join you at research visits, such as a spouse, family member, or friend?” [18]. At present, it is unknown how often individuals screen-fail for clinical trial participation because they cannot or will not identify a study partner.

In many AD trials, as many as two thirds of participants enroll with a spouse or domestic partner as their study partner (“spousal study partners”) whereas only a quarter of participants enroll with an adult child [19]. In a study of recruitment in multinational AD clinical trials, although there was heterogeneity, more than 70% of participants enrolled with a spousal study partner in North America, Western Europe, Israel, Australia, and South Africa [20]. Yet many, if not most, AD caregivers are adult children [12]. The disproportionately high representation of spousal study partners is striking: individuals *without* a spouse or domestic partner comprise the majority of the population of potential research volunteers [21, 22].

Overall differences between the general AD population and the population enrolled in AD trials suggest that barriers to recruitment of adult children or friends as study partners (“non-spousal study partners”) significantly shape the population under study [23]. For example, in the US, the proportion of minorities among older adults is increasing [24], and African-Americans and Hispanics are at higher risk than Caucasians for developing AD [12]. Yet racial minorities are significantly under-represented in most AD trials [25]. Minority participants who do enroll are more likely to enroll with a non-spousal study partner [10, 26]. Diversity of trial samples must be improved in order to ensure more thorough understanding of sub-population treatment effects and to reduce disparities in the burden of AD.

Facilitating the participation of non-spousal study partners in particular may allow AD researchers to realize a twofold benefit: faster accrual and greater generalizability. Below, we examine the factors that influence a study partner’s decision *not* to participate and then we propose solutions that address these factors.

## Factors influencing study partners’ participation

At least three factors may explain the discrepant participation rates of spousal and non-spousal study partners.

First, a person who participates in an AD trial as a study partner experiences opportunity costs, a term describing the value of the next best option faced by the study partner, such as working or caring for someone else instead of being in a trial. Over half of adult-child caregivers are in the workforce [27], and around a quarter care for a minor child or children in addition to their aging parent [12]. Therefore, the opportunity costs of research participation are likely to be higher for an adult child than for a spouse or domestic partner with more limited work and familial responsibilities.

Second, non-spousal study partners appear to have less favorable attitudes toward research. Interviews with more than 100 AD caregivers showed that spouses had greater willingness to participate in a 21-month-long AD dementia clinical trial than adult children [21]. Spousal caregivers expressed greater trust in research than adult-child caregivers and more positive attitudes toward research, which were associated with greater willingness to enroll.

Third, the decision *not* to participate in research is often made unilaterally by the caregiver [28, 29] because they doubt that the intervention will be effective, fear side effects for the patient, or wish to avoid increasing the patient’s medical burden [30, 31]. These perceived downsides to participation are apparently seen as relatively weightier by adult children than by spouses or domestic partners [23]. These data suggest that the burdens of participating in research may appear relatively greater and the benefits more remote for non-spousal compared with spousal study partners.

## A path forward

A holistic effort to address the AD recruitment crisis requires taking seriously the work of being a study partner.

### Removing obstacles to study partners’ participation

To the extent possible within the limits of methodological rigor and scientific validity, investigators must collaborate with older adults and their prospective study partners to ensure that AD trials are designed to be palatable to dyads. For example, sponsors might adopt adaptive study designs in order to shorten trials or reduce sample sizes [2]. Combining phases and choosing appropriate primary endpoints may lead to more efficient trials. Smaller trials require less recruitment, and one might expect that recruitment is easier in shorter trials [23]. Or investigators might increase the proportion of subjects allocated to the experimental treatment if a higher likelihood of randomization to the experimental treatment is sufficiently desirable to increase enrollment rates. This approach, however, requires larger sample sizes [32] and as the probability that a subject receives active medication increases above chance (50-50), the more likely the trial will experience bias from subjects’ and investigators’ belief that the subjects are on medication [33]. Improvement of clinical trial design is just one step, however, and we should not fail to use other tools at our disposal to remove real and perceived obstacles to study partner participation.

AD study visits typically occur during weekday business hours. Accompanying the participant to study visits may require the study partner to rearrange her work schedule or take time off. The opportunity costs of research participation could be reduced by offering study visits at night or on weekends. Other ways include addressing travel to the study site. Study partners’ willingness to participate in an AD trial significantly increases when the study partner is offered home visits or an optional car service to transport the participant and study partner to study visits [30]. Some trials are already using this insight and offering transportation to study visits via the ride-sharing application Lyft [34].

The study partner could also be allowed to participate remotely, for example, by using Skype or a similar application to report for safety checks (i.e., when no study outcome is being assessed) or even to complete study instruments. Evidence suggests that it is possible to carry out accurate and reliable cognitive assessments via videoconferencing and telemedicine [35]. Offering remote participation may improve willingness to enroll in AD trials, as has been shown in Parkinson’s disease trials [36].

### Reimbursing study partners’ out-of-pocket costs

AD research participation has out-of-pocket costs (for example, mileage and parking) that can be reimbursed [37]. These fixed costs exact a relatively greater burden on persons experiencing financial strain than on persons who are wealthier. A recent study in American patients with cancer found that addressing this burden by using a financial assistance program that reimbursed all travel and lodging expenses for individuals with incomes of not more than 400% of the federal poverty level (FPL) increased clinical trial participation [38]. The FPL is an income measure issued annually by the US Department of Health and Human Services; in 2018, the FPL is $12,140 for individuals [39]. Reimbursement that reduces the financial burden of the fixed costs to AD trial participation should be tested to assess whether it promotes fairer access to trials for lower-income patients.

### Compensating study partners for time and effort

It is widely accepted that treating people fairly means adequately compensating them for their work. The social value of an effective disease-modifying intervention for AD is clear, and the necessity of the study partner’s contribution to making that breakthrough is well established [11]. Thus, study partner compensation for the time and effort of research participation should be the default [40].

What constitutes fair compensation for study partners? The wage-payment model posits that research participation requires time and effort with little need for specialized skills [41]. Adopting this model to compensating study partners would mean payment of a fairly low, standardized hourly wage (that is, at or close to the federally mandated minimum wage). Notably, such compensation reflects the value of the study partner’s research participation work and not the study partner’s wages, or forgone wages, in daily life.

### Offering incentives to study partners

Incentives are an offer of payment in excess of what would be required to reimburse or compensate. Incentives are more controversial than either reimbursement or compensation. Incentives can, however, be ethically permissible if they are effective in preventing studies from falling short of recruitment targets and, as a result, from being underpowered or terminating early [9, 40]. No data exist to show whether incentives to study partners improve recruitment for AD clinical trials, but evidence from survey research convincingly shows that larger offers of payment are more effective at increasing survey participation [42].

Incentives—by design—encourage people to do something they might not otherwise do, such as participate in research. A common concern, however, is that excessive incentives may distort decision making, causing the recipient to do something unreasonable [43]. Although the study partner does work in an AD study, participation poses no risk to her. Rather, the person with AD is the object of study and bears research-associated risks. Therefore, participation is unlikely to be unreasonable for the study partner, but it could be unreasonable for the older adult. A useful comparison can be drawn to pediatric research [44]. Because children cannot legally consent for themselves, a parent or guardian must decide whether to enroll them in research. The concern when payment is offered to families is that a desire for financial gain may cause parents to agree to research participation they would otherwise decline as contrary to their child’s interests [43]. Analogously, incentive payments might unacceptably distort study partners’ surrogate decision-making on behalf of the older adult. This concern is not, however, relevant in preclinical AD trials, where prospective participants necessarily retain the capacity to give informed consent, and will be less worrisome in MCI trials if participants can actively participate in decision making.

Incentives might disproportionately incentivize dyads of lower socioeconomic status (SES) to participate, but evidence is inconclusive that people with lower incomes are more likely to be encouraged to participate in research when offers of incentive payments are higher [37]. However, even if we accept that low-SES dyads are more sensitive to incentives than their higher-SES counterparts, that is not the same as saying that low-SES dyads are more prone to *distorted* decision making. Rather, they may reasonably make trade-offs between their financial and non-financial interests when deciding to participate in research.

Finally, even if incentives do not distort study partners’ decision making, they may introduce selection bias [45]. Dyads motivated primarily by monetary gain may be less reliable: they may be less likely to adhere to study procedures, report side effects, or attend required clinic visits. Empirical research is needed to determine whether this is, in fact, true.

Without data that show incentives to study partners cause such problems, a reasonable approach is to offer relatively modest incentives to ensure sufficient enrollment in important research [43]. Independent review by an institutional review board helps ensure that participation is reasonable for prospective research participants [46]. In circumstances where concerns about offering monetary incentives to study partners are insurmountable, it may be possible to offer non-monetary incentives such as gratis respite care.

### Accounting for study partners’ ongoing workforce participation

To improve study partner recruitment, researchers could immediately take the steps described above. Researchers cannot, however, be expected to make up for social injustice—namely, an over-reliance on unpaid informal caregiving. There are, however, steps by which other stakeholders could address this injustice and facilitate study partner recruitment.

More than two thirds of working caregivers need to rearrange their work schedule, decrease their work hours, or take unpaid leave to meet caregiving responsibilities [47]. When we ask these individuals to participate in research, we are asking them to take on additional responsibilities that will further affect their workforce participation. For some caregivers, this is not feasible.

In the US, the Family and Medical Leave Act of 1993 (FMLA) provides certain employees with up to 12 weeks of *unpaid*, job-protected leave annually to fulfill caregiving responsibilities. Yet only about 60% of the workforce is eligible for these protections [48]. The FMLA and its analogues in other countries should be amended to encompass more workers and to reduce the risk of job loss for employed study partners who wish to contribute to AD research. Although other countries have more generous family and medical leave policies, they do not generally protect time for research participation.

Additionally, public and private employers committed to finding better treatments for AD [49] could offer *paid* time off for employees participating in AD clinical research as study partners. From the study partners’ perspective, paid time off will mitigate the financial impact of participation. From the employers’ perspective, accelerating AD research will, in the long run, reduce the significant costs of AD to businesses, which include absenteeism, productivity losses, and replacement costs as workers struggle with the responsibilities of AD caregiving as well as health and long-term care expenditures [50]. Of course, business costs associated with offering paid leave will predominate in the short term. Another limitation of this approach is that paid time off is generally concentrated among high-income workers [51] and therefore may not have the desired impact on diversity of research participants.

### Studying the study partners

The strategies described above ought to be studied to show that, in fact, they do improve overall recruitment rates and generalizability. Additional questions about study partners also require study:

1. *Do these methods increase the representativeness of AD study participants?* The degree to which recruitment barriers are driven by factors associated with minority race rather than non-spousal study partner status is unknown. Some research suggests that the largest deterrents to minority recruitment are procedural rather than cultural [52]. Others have found that race is independently associated with willingness to participate in research [53], and institutional factors may be important [54].
2. *Compared with spouses, do non-spousal study partners have distinct motivations to enroll in research?* As described above, non-spousal study partners may have less favorable attitudes toward research than spousal study partners and different motivations for participating in AD research. Better understanding of these differences could lead to targeted educational interventions as well as specifically tailored advertisements and recruitment messages that maximize enrollment of non-spousal study partners. Messaging may also need to vary between sites in multinational trials.
3. *To what extent are adult children and friends capable of fulfilling study partner responsibilities?* Study partner characteristics may be associated with the accuracy of their reports on measures that establish the efficacy of an AD intervention. For example, spouses may provide more accurate data than non-spousal relatives on patient cognition [55]. More information is needed to instruct investigators on how best to address this possible bias.
4. *Can the study partner role be shared among multiple individuals?* Depending on the extent of cognitive impairment and the demands of the research protocol, study partners function in a number of different capacities. Yet it is not necessarily the case that one person must (or even should) take on the multiplicity of roles. For instance, it may be that a spouse or adult child legally must provide surrogate consent for research participation for an adult with dementia but that others, such as a paid caregiver, could reasonably serve as an informant on questions of function and cognition. Or it may be that several individuals could share a single role—for example, several adult children might take turns accompanying the participant to visits or even acting as informant, although inherent risks to data integrity would need to be addressed in the setting of the latter.
5. *Do interventions designed to increase recruitment also increase retention rates and reduce the problem of missing data?* Attrition is a problem of ethical and scientific significance in clinical research. In AD trials, completion rates are lower among non-spousal dyads [10], and in one natural history study, informant replacement occurred more frequently in participants lacking a spouse [56]. Participant dropouts lower statistical power and create confounding if dropouts are non-random. Modern statistical analysis tools—such as multiple imputation—can be used to address missing data, but they have important conceptual limitations. Thus, a principled strategy to address missing data requires careful trial design and conduct to limit the amount of missing data *in addition to* thoughtful use of statistical analysis tools [57]. Many of the design and recruitment strategies discussed above could reasonably be hypothesized to improve both recruitment and retention rates, as a complement to statistical approaches to address missing data.
6. *Which approaches to enhancing study partner participation in AD research are most cost-effective?* The cost of developing a disease-modifying drug for AD is estimated to approach $5.7 billion, and clinical trials now comprise the costliest aspect of drug development [58]. Prior work has shown that it can be more expensive to recruit caregivers depending on their demographic characteristics [59], and costs are further compounded when a dyad is needed [60]. It is essential to determine whether the additional costs of offering payment or other changes within AD trials, such as those described above, would lower costs from more efficient recruitment and lower dropout rates at a level sufficient to justify their widespread adoption.

## Conclusion

Given the devastating effects of AD, it is imperative that AD trials expedite recruitment and enroll a more representative sample of participants in order to speed discovery and approval of AD-modifying treatments. To reach the global goal of having a treatment or cure for AD by 2025, we need the best science and statistical methods but also innovative means of ensuring adequate dyad recruitment.
